# Supraphysiological Testosterone Differentially Regulates Aortic Atheroma and Cardiac Remodelling in the Testicular Feminised Mouse

**DOI:** 10.3390/cells15181633

**Published:** 2026-09-09

**Authors:** Daniel M. Kelly, Joanne E. Nettleship, Marta M. Gillett, T. Hugh Jones

**Affiliations:** 1 Biomolecular Research Centre, Sheffield Hallam University, Sheffield S1 1WB, UK; m.gillett@shu.ac.uk; 2 Division of Clinical Medicine, University of Sheffield, Sheffield S10 2RX, UK; joannenettleship@hotmail.com (J.E.N.); hugh.jones@nhs.net (T.H.J.); 3 Centre for Diabetes and Endocrinology, Barnsley Hospital NHS Foundation Trust, Barnsley S75 2EP, UK

**Keywords:** testosterone, atherosclerosis, androgen receptor (AR), cardiac hypertrophy, testosterone replacement therapy (TTh)

## Abstract

**Highlights:**

**What are the main findings?**

- Physiological and supraphysiological testosterone reduces diet-induced aortic atheroma formation in androgen receptor-deficient mice, consistent with mechanisms beyond classical androgen receptor signalling.
- Supraphysiological testosterone promotes early cardiac hypertrophic remodelling in androgen receptor-intact mice.

**What are the implications of the main findings?**

- Testosterone exerts divergent cardiovascular effects, limiting early atherogenesis while promoting androgen receptor-dependent cardiac remodelling.
- The findings support maintaining physiological testosterone concentrations to maximise cardiovascular benefit while avoiding unnecessary androgen excess.

**Abstract:**

The cardiovascular actions of testosterone remain controversial, particularly regarding the safety of testosterone replacement therapy (TTh) in hypogonadal men. We investigated the effects of sustained supraphysiological testosterone exposure on aortic atherogenesis and cardiac remodelling in the testicular feminised (Tfm) mouse, a model of functional androgen receptor (AR) deficiency. Male Tfm mice and AR-intact XY littermate controls were fed a cholesterol-enriched diet for 28 weeks and received fortnightly intramuscular injections of saline or supraphysiological testosterone, alone or in combination with fulvestrant (oestrogen receptor antagonist) or anastrozole (aromatase inhibitor). Aortic lipid deposition was quantified by Oil Red O staining, while cardiac remodelling was assessed by heart weight, cardiomyocyte cross-sectional area and myocardial gene expression. Supraphysiological testosterone significantly reduced aortic fatty streak formation in Tfm mice compared with saline-treated controls (1.25 ± 0.36% vs. 2.85 ± 0.37%, *p* < 0.01), an effect preserved following fulvestrant or anastrozole treatment, consistent with mechanisms that do not require classical AR or oestrogen receptor signalling. No additional reduction in aortic lipid deposition was observed in AR-intact XY littermates. In contrast, supraphysiological testosterone increased heart weight, cardiomyocyte size and expression of hypertrophic markers exclusively in XY mice, with no evidence of cardiac remodelling in Tfm mice. Collectively, these findings demonstrate divergent tissue-specific cardiovascular actions of testosterone, whereby supraphysiological exposure promotes AR-dependent cardiac remodelling without conferring additional vascular benefit, supporting maintenance of physiological testosterone concentrations during TTh.

## 1. Introduction

Testosterone is increasingly recognised as an important regulator of cardiometabolic health, influencing processes including carbohydrate and lipid metabolism, inflammation, vascular function and tissue remodelling [1,2,3,4]. Despite considerable interest in the role of testosterone in cardiovascular disease, controversy has persisted. Early observational studies reported an increased risk of cardiovascular events during testosterone therapy [5,6,7], although many of these findings were subsequently challenged on methodological grounds [8,9,10]. More recently, the TRAVERSE trial demonstrated that testosterone replacement therapy was non-inferior to placebo with respect to major adverse cardiovascular events in appropriately selected hypogonadal men, providing reassurance regarding the cardiovascular safety of physiological testosterone replacement [11]. These findings have since been incorporated into recent international position statements, which conclude that current evidence does not support an increased risk of major adverse cardiovascular events with physiological testosterone replacement when appropriately prescribed, while recognising that important uncertainties remain in specific patient groups and with supraphysiological androgen exposure [12,13]. Although the long-term effects of testosterone therapy remain incompletely defined, epidemiological and registry studies suggest that restoration of testosterone concentrations towards the physiological range in hypogonadal men may be associated with favourable cardiometabolic outcomes [11,14]. In contrast, exposure to supraphysiological androgen concentrations, such as those observed with anabolic-androgenic steroid abuse, has been associated with adverse cardiovascular remodelling and impaired cardiac function [15]. Accumulating evidence suggests that testosterone exerts important actions within the vasculature and myocardium that may influence the development of both atherosclerosis and cardiac remodelling [1].

Epidemiological and clinical studies have consistently demonstrated an association between low endogenous testosterone concentrations and an adverse cardiometabolic phenotype. Men with proven coronary artery disease and greater than 50% stenosis of at least one artery have lower testosterone levels than men with normal coronaries [16]. Furthermore, prospective studies have demonstrated associations between lower testosterone concentrations and increased long-term mortality risk, highlighting the potential prognostic relevance of endogenous testosterone status [14]. Testosterone deficiency is linked to obesity, insulin resistance, type 2 diabetes and dyslipidaemia [17], while lower circulating testosterone concentrations have also been associated with increased carotid intima-media thickness [18], greater atherosclerotic burden and an elevated risk of cardiovascular mortality [19,20]. Experimental androgen deprivation, as occurs during androgen deprivation therapy for prostate cancer, similarly promotes metabolic dysfunction and increases cardiovascular risk [21]. TRAVERSE, the largest randomised placebo-controlled cardiovascular outcomes study of testosterone therapy conducted to date, demonstrated no increase in major adverse cardiovascular events in hypogonadal men receiving TTh [11]. The mechanisms through which testosterone influences vascular pathology and cardiac remodelling, however, remain incompletely understood.

Preclinical models provide an opportunity to investigate the vascular and cardiac actions of testosterone under controlled experimental conditions and independent of many of the confounding factors present in clinical studies. The testicular feminised (Tfm) mouse, which lacks functional androgen receptors (AR), has provided important insights into the role of androgen signalling in cardiovascular disease. Using this model, we have previously demonstrated that physiological testosterone replacement attenuates aortic fatty streak formation despite the absence of functional AR signalling, highlighting the complexity of testosterone action within the vasculature [22]. However, while these findings suggest that testosterone may exert atheroprotective effects through non-classical pathways, considerably less is known regarding the cardiovascular consequences of sustained exposure to supraphysiological testosterone concentrations. In addition to its vascular actions, testosterone has been implicated in the regulation of cardiac structure and function. Whilst physiological testosterone concentrations appear to be important for maintaining normal cardiovascular health, evidence from athletes and anabolic-androgenic steroid users suggests that chronic supraphysiological androgen exposure may promote cardiac hypertrophy and adverse myocardial remodelling [23].

Defining the vascular and cardiac responses to excessive androgen exposure may provide important mechanistic insights into the therapeutic window of testosterone action and inform strategies for the safe optimisation, monitoring and titration of testosterone therapy. Therefore, the present study utilised the Tfm mouse and AR-intact littermate controls to determine the effects of sustained supraphysiological testosterone exposure on aortic fatty streak formation and cardiac remodelling, and to establish the relative contribution of AR-dependent and AR-independent pathways to these processes.

## 2. Materials and Methods

### 2.1. The Tfm Mouse

The Tfm mouse, which carries an X-linked single base-pair deletion in the gene encoding the classical AR, was used in this study [24]. This mutation causes a frameshift in the AR mRNA, resulting in a premature stop codon in the amino-terminal region and production of a truncated receptor that lacks both DNA- and steroid-binding domains [25]. Consequently, Tfm mice are completely AR-insensitive and display a female-like phenotype. In addition, circulating testosterone levels are markedly reduced due to a deficiency of 17α-hydroxylase in Leydig cells, impairing steroidogenesis [26,27]. Mice were bred and maintained as previously described [28,29], and all procedures were conducted under UK Home Office project licences PPL 40/2227 and PPL40/3165, in accordance with the Animals (Scientific Procedures) Act 1986, and after local ethical review by the University of Sheffield ethical review committee.

### 2.2. Determination of a Reproducible Dosing Regimen to Replace Testosterone at Supraphysiological Levels in the Tfm Mouse and XY Littermates

In order to assess the impact of testosterone therapy upon fatty streak formation in the Tfm mouse, the establishment and maintenance of a dosing regimen to replace testosterone at supraphysiological concentrations was undertaken. Consequently, eight-week-old Tfm mice (*n* = 35) and XY littermates (*n* = 24) received a single 10 µL intramuscular injection of 250 mg/mL testosterone (Sustanon 250^®^, Bayer AG, Leverkusen, Germany) providing a dose of 125 mg/kg. Mice were then sacrificed at serial intervals at one, two, four, seven, 10 or 14 days post injection via a Home-Office-approved Schedule 1 method. To ensure reproducibility of this pharmacokinetic profile, additional Tfm (*n* = 20) and XY (*n* = 23) mice received a second injection on day 14 and were sacrificed on days 15, 16, 18, 21 or 28 via a Home-Office-approved Schedule 1 method. This regimen was compared with a physiological dose of 10 µL intramuscular injection of 100 mg/mL testosterone (Sustanon 100^®^, Bayer AG, Leverkusen, Germany) providing a dose of 50 mg/kg, with the same fortnightly injection schedule and analysis points.

### 2.3. Experimental Design

Eight-week-old Tfm and XY littermate mice were fed either a high-fat, high-cholesterol diet containing 42% butterfat, 1.25% cholesterol, and 0.5% cholate (Special Diet Services, Essex, UK) ad libitum for a period of 28 weeks to induce atheroma. Animals were weighed on a weekly basis. To assess the impact of testosterone on fatty streak formation in the Tfm mouse, separate seven-week-old mice were randomly assigned to either a placebo group (*n* = 14) receiving fortnightly intramuscular injections of 10 μL of saline, or a testosterone group (*n* = 14) receiving fortnightly intramuscular injections of physiological testosterone (Sustanon 100^®^), previously described [28,29], or supraphysiological testosterone (Sustanon 250^®^, as established in Section 2.2). Animals were allocated to experimental groups prior to treatment without selection based on individual phenotype or experimental outcome. No formal randomisation sequence was used. Potential confounding factors, including cage location and the order of treatment administration or tissue collection, were not formally controlled or randomised. Tfm mice were compared with XY littermate mice receiving placebo (*n* = 14) or Sustanon 250^®^ (*n* = 12).

At eight weeks of age, additional Tfm mice (*n* = 15) were randomly separated into two further experimental groups. Each group received fortnightly intramuscular injections of 10 µL Sustanon 250^®^ in conjunction with either an intramuscular injection of 30 µL of 50 mg/mL oestrogen receptor-α (ERα) antagonist fulvestrant (Faslodex^®^ – AstraZeneca, Cheshire, UK: 15 times the human dose) (*n* = 8), or with 10 mg/kg/day of the aromatase inhibitor anastrozole (Arimidex^®^ – AstraZeneca, Cheshire, UK: 10 times the human dose) in the drinking water for the duration of the experimental period (Tfm, *n* = 7). Group sizes were determined a priori using power calculations based on preliminary pilot data, in consultation with statistical support at the University of Sheffield, to ensure sufficient statistical power while minimising animal use. Treatment allocation was known to the investigators during animal allocation, treatment and tissue collection. Following tissue collection, animals and samples were coded, and all subsequent experimental measurements and outcome assessments were performed with investigators blinded to treatment allocation. Samples were decoded only after completion of the analyses.

### 2.4. Tissue Collection

At the end of the relevant feeding or dosing period, whole blood was collected from the abdominal cavity following midline sternotomy and severance of the thoracic aorta and, following centrifugation, serum samples were frozen at −80 °C. Following the opening of the rib cage, the heart with thoracic aorta attached was carefully dissected free from the adventitia and perfused with physiological saline solution to remove any blood clots. Thoracic aortas were removed directly proximal to the initial curvature of the aortic arch and whole hearts were subsequently weighed. The basal half of the ventricles and the ascending aorta were removed, and the upper half of the heart with the aortic root attached was embedded in optimum cutting temperature compound (Bright Instrument Company Ltd., Huntingdon, UK) and snap-frozen in liquid nitrogen-cooled isopentane. This process was replicated for ventricular tissue. Tissues were processed for both histological and gene expression analyses and were archived for future analysis. Analyses were made on individual samples.

### 2.5. Measurement of Total Testosterone

Serum total testosterone (DRG Instruments GmBH, Marburg, Germany) was measured in duplicate via ELISA (measurement range 0.2–16 ng/mL).

### 2.6. Oil Red O Quantification of Fatty Streak Formation

Transverse frozen sections (8 μm-thick) of the aortic root with valves (approximately 50 per mouse) were cut on a cryostat, and five sections (every tenth section) of the aortic root were used per animal for Oil Red O (ORO) staining as described by Nettleship et al. [28]. Lipid-stained areas were quantified with ImageJ software (v1.54, NIH-ImageJ, National Institutes of Health, Bethesda, MD, USA) and were expressed as a percentage of the intimal plus medial area, and the mean ± SEM from the five sections was calculated for each animal.

### 2.7. Cardiomyocyte Cross-Sectional Area (CSA)

Cardiomyocyte cross-sectional area was quantified from three to five transverse sections (8 μm-thick) per heart obtained from the mid-apical region of the left ventricle. Sections were stained with H&E using standard histological techniques and images were then captured using an Olympus BX60 microscope (Olympus Optical Co., Ltd., Tokyo, Japan) and quantified using analysis software (ImageJ v1.54) with 10–18 cardiomyocytes analysed per section, yielding approximately 40 cells per animal with *n* = 6 per group. CSA data are presented as individual measurements, with statistical analyses performed using group means of *n* = 6.

### 2.8. Quantitative Analysis of mRNA

Total RNA was extracted from approximately 10–30 mg of snap-frozen tissue, reverse-transcribed to cDNA using a commercial reverse-transcription kit (Qiagen Ltd., Crawley, UK), according to the manufacturer’s instructions.

Quantitative real-time PCR was performed using pre-designed TaqMan Gene Expression Assays (Thermo Fisher Scientific, Waltham, MA, USA) and TaqMan Universal PCR Master Mix on an Applied Biosystems 7500 Fast Real-Time PCR System. Gene expression was assessed for the following targets: Nppa (encodes atrial natriuretic peptide [ANP]): Mm01255747_g1, Nppb (B-type natriuretic peptide [BNP]): Mm01255770_g1, Acta1 (α-skeletal actin): Mm00808218_g1, Myh7 (β-myosin heavy chain [β-MHC]): Mm00600555_m1, Tagln (transgelin [SM22α]): Mm00441661_g1, and Gsk3b (glycogen synthase kinase-3β [GSK3β]): Mm00444911_m1, with ribosomal protein L4 (Rpl4: Mm05781370_g1) used as the endogenous reference gene, selected as the most stable based on evaluation of a commonly used panel of housekeeping genes (Gapdh: Mm99999915_g1, Actb: Mm02619580_g1, Rpl13a: Mm05910660_g1, Hprt: Mm03024075_m1).

All reactions were performed in triplicate in 96-well plates under standard cycling conditions: 50 °C for 2 min, 95 °C for 10 min, followed by 40 cycles of 95 °C for 15 s and 60 °C for 1 min. Relative gene expression was calculated using the comparative Ct (2^−ΔΔCt^) method, normalised to Rpl4 and expressed relative to the untreated XY littermate control group.

### 2.9. Statistical Analysis 

Baseline numbers for individual analyses were determined by power calculations from preliminary and previous data [28]. No specific exclusion criteria were established a priori for animals, tissue samples or individual data points. All available samples were included in analyses where technically feasible. For individual tissue analyses, subsets of animals were selected from the experimental groups according to tissue availability and experimental requirements; these subsets were selected without regard to experimental outcome. Thus, the number of animals analysed varied between experiments and is reported for each individual analysis. No animals or data points were excluded on the basis of the experimental outcome or observed phenotype.

Results are presented as mean ± SEM. Differences in normally distributed continuous variables were compared using one-way analysis of variance (ANOVA), with corrections for multiple comparisons made using Tukey’s post hoc test. Differences in non-normal data were compared using the Kruskal–Wallis test followed by the Dunn’s post hoc correction for multiple comparisons. Statistical significance was accepted at *p* ≤ 0.05. Exploratory pairwise comparisons were additionally performed using unpaired two-tailed Student’s *t*-tests for normally distributed variables or Mann–Whitney U tests for non-normally distributed variables, to provide preliminary insight into specific group differences and guide future hypothesis-driven experiments.

## 3. Results

### 3.1. Determination of a Reproducible Dosing Regimen to Replace Testosterone at Supra-Physiological Levels in the Tfm Mouse and XY Littermates

**Figure 1:**
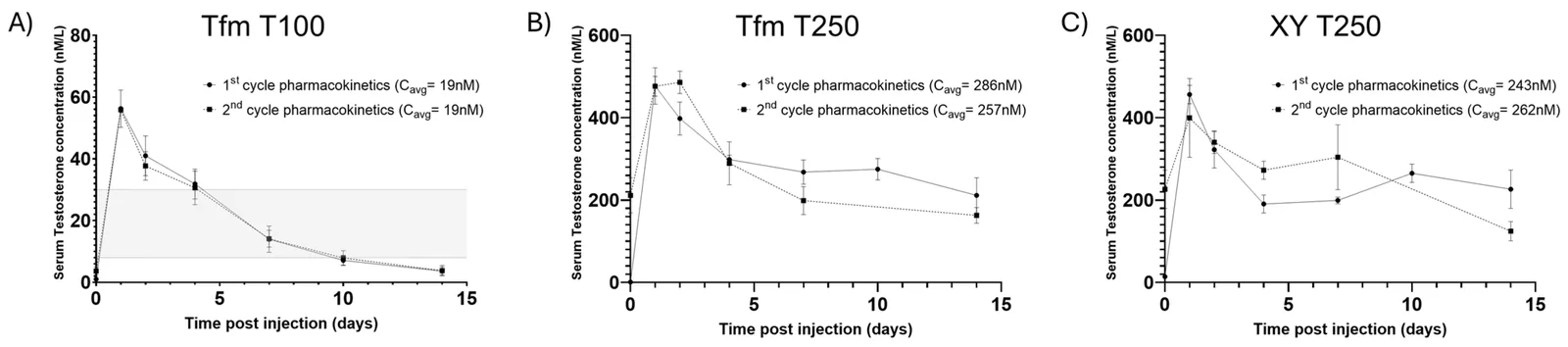
Pharmacokinetic Profiles of Testosterone in Tfm and XY Control Mice: Serum testosterone concentration kinetics over a four-week period following intramuscular injections every two weeks. (**A**) Serum testosterone concentrations in Tfm mice administered physiological T100 doses. (**B**) Tfm mice administered supraphysiological T250 doses, and (**C**) XY littermate controls administered supraphysiological T250 doses. Each panel displays overlaid serum testosterone concentration–time profiles across the fourteen-day inter-injection interval for phase 1 (solid line) and phase 2 (dashed line) of the dosing schedule, with the corresponding time-average concentration (Cavg) plotted for each treatment week. Values are presented as mean ± SEM (*n* = 8 per group).

Physiological T100 dosing in Tfm mice produced serum testosterone concentrations within the wild-type physiological range, consistent with previously published data from this model [22,28]. Following the initial 10 µL intramuscular injection of 100 mg/mL testosterone, serum testosterone concentrations rose sharply from baseline (2.2 ± 1.2 nM in untreated Tfm mice) into the supraphysiological range (>20 nM based on wild-type reference values), remained elevated through approximately day 4, declined to within the physiological range by day 7, and fell below the physiological range by day 10 prior to the second injection (Figure 1A). The second-phase dosing interval followed the same pattern, with time-average concentrations (C_avg_) of 19 nM for both two-week phases.

Supraphysiological T250 dosing (250 mg/mL) in Tfm mice produced a rapid rise in serum testosterone to a peak of 477 nM, with concentrations maintained in the supraphysiological range across the full 28-day study period, with C_avg_ values of 286 nM and 257 nM in phase 1 and phase 2, respectively (Figure 1B). A comparable concentration–time profile was observed in XY littermates receiving the same 250 mg/mL dose, again with sustained supraphysiological concentrations throughout the 14-day dosing intervals and C_avg_ values of 243 nM and 262 nM for phase 1 and phase 2, respectively (Figure 1C). Consistent pharmacokinetic patterns across the two-week injection interval suggest sustained delivery efficacy despite the distinct genetic background and inherent androgen sensitivity variations between the groups.

### 3.2. Determination of the Effect of Supraphysiological Testosterone Therapy and the Influence of the AR upon Atheroma Formation

**Figure 2:**
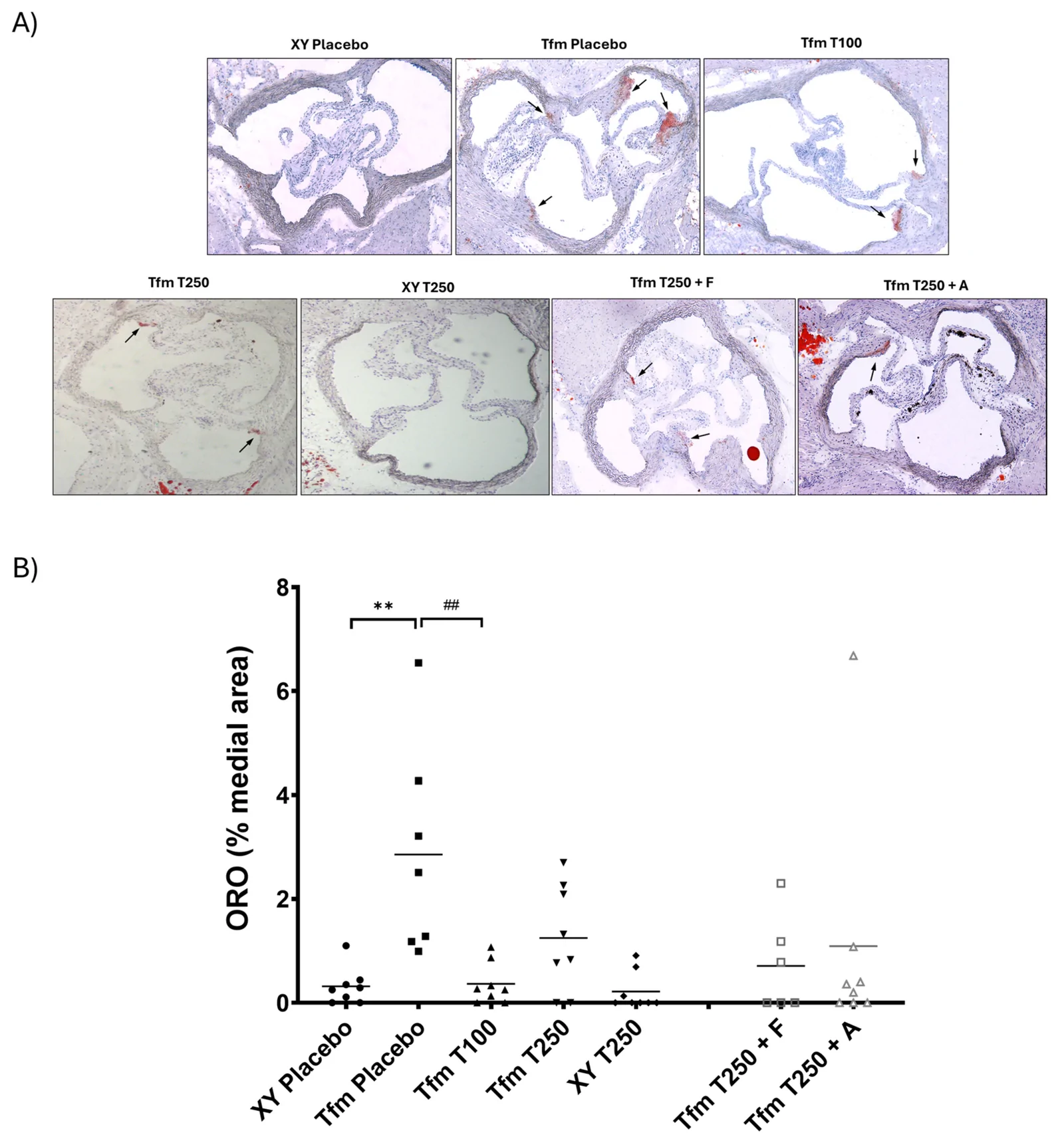
Effect of physiological and supraphysiological testosterone on aortic root lipid accumulation in Tfm mice and XY littermate controls. (**A**) Representative photomicrographs of Oil Red O-stained cross-sections of the aortic root showing neutral lipid deposition in the intimal–medial layer across the treatment groups (red staining indicated by arrows): XY placebo, Tfm placebo, Tfm T100, Tfm T250, XY T250, Tfm T250 + F (fulvestrant), and Tfm T250 + A (anastrozole). Representative images acquired at 40× total magnification (4× objective, 10× eyepiece). (**B**) Quantitative analysis of ORO-positive staining expressed as a percentage of the total intimal–medial cross-sectional area per section, with mean values calculated from five serial sections through the aortic root (*n* = 8 animals per group). Data are presented as mean ± SEM. Statistical significance was determined by one-way ANOVA with Tukey’s post hoc multiple comparisons test; significance levels are denoted by ** *p* < 0.01 vs. XY placebo, ## *p* < 0.01 vs. Tfm placebo.

ORO staining of the aortic root revealed markedly increased intimal–medial neutral lipid deposition in placebo-treated Tfm mice relative to the XY placebo controls (*p* < 0.05; Figure 2B), consistent with the representative photomicrographs shown in Figure 2A, which are indicative of increased fatty streak formation.

In Tfm mice, physiological testosterone replacement (T100) substantially reduced % ORO-positive area within the intimal–medial layer versus the Tfm placebo group (*p* < 0.01), with values comparable to the XY placebo controls (0.365 ± 0.14% vs. 0.318 ± 0.12%, respectively). A comparable reduction was observed with supraphysiological testosterone (T250) in Tfm mice although this was not statistically significant (vs. Tfm placebo, *p* < 0.179), and no significant difference was apparent between T100 and T250 groups. In XY littermates, T250 had no added effect on ORO staining compared to the XY placebo, and no significant difference was observed compared with the Tfm T100 or Tfm T250 groups.

The degree of lipid deposition within the aortic root of Tfm mice receiving supraphysiological testosterone therapy in conjunction with fulvestrant or anastrozole was statistically similar to the level reported above in Tfm mice receiving supraphysiological testosterone therapy alone; 0.71 ± 0.38% and 1.09 ± 0.81%, respectively, versus 1.246 ± 0.36% (both *p* = ns), and the two intervention groups did not differ significantly from each other (Figure 2B).

### 3.3. Supraphysiological Testosterone Induces Androgen Receptor-Dependent Cardiac Hypertrophic Remodelling

**Figure 3:**
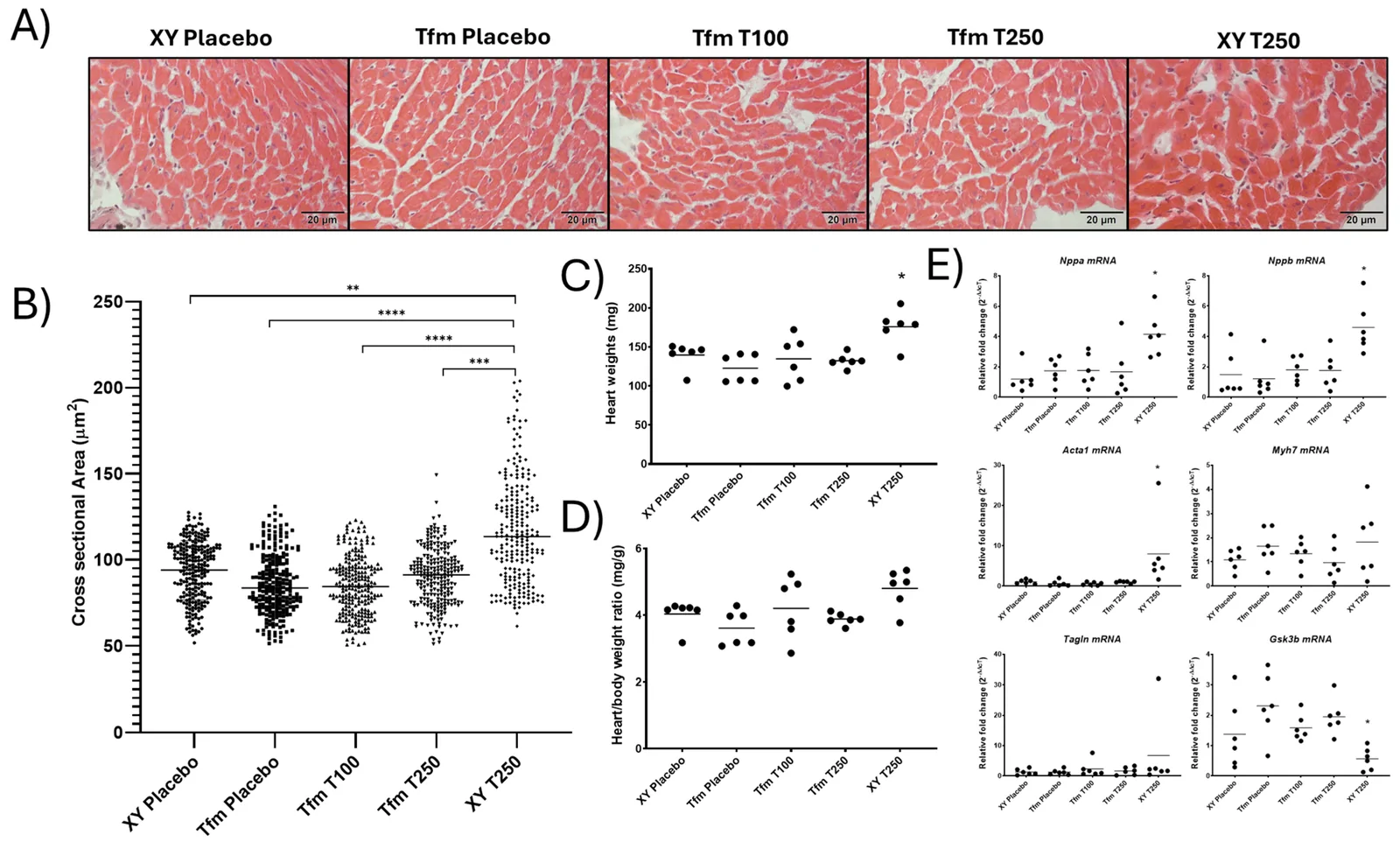
Testosterone effects on cardiomyocyte size, heart weight, and hypertrophy markers in Tfm and XY mice. (**A**) Representative photomicrographs of left ventricular cross-sections showing cardiomyocyte cross-sectional area (CSA) of treatment groups. (**B**) Quantification of cardiomyocyte CSA, with data presented as individual measurements and statistical analyses performed using group means, *n* = 6. (**C**) Heart weight at endpoint across treatment groups. (**D**) Heart weight normalised to body weight (HW:BW ratio). (**E**) Relative expression of cardiac hypertrophy marker genes (as specified in the panel) across treatment groups, determined by real-time qPCR and expressed relative to the Rpl4 reference gene. Scale bars: 20 µm in panel A. Data in (**B**–**E**) are presented as mean ± SEM (*n* = 6 per group). * *p* < 0.05, ** *p* < 0.01, *** *p* < 0.001, **** *p* < 0.0001.

Representative H&E-stained left ventricular sections demonstrated increased cardiomyocyte cross-sectional area in XY littermates receiving supraphysiological testosterone (T250) compared with XY placebo controls, whereas no obvious differences were observed between treatment groups in Tfm mice (Figure 3A). Quantitative image analysis confirmed a significant increase in cardiomyocyte cross-sectional area in XY T250 mice compared with XY placebo controls (118.4 vs. 93.7 μm^2^, *p* < 0.05; Figure 3B). Cardiomyocyte cross-sectional area did not differ significantly among placebo-, physiological testosterone- (T100), or supraphysiological testosterone-treated (T250) Tfm mice.

Consistent with these findings, absolute heart weight was significantly increased following supraphysiological testosterone treatment in XY littermates compared with XY placebo controls (175.9 vs. 139.5 mg, *p* < 0.05; Figure 3C). Body weight did not differ significantly between experimental groups at study endpoint (all *p* = ns). Consequently, normalisation of heart weight to body weight attenuated this difference, and the heart weight: body weight ratio was not significantly altered between groups (Figure 3D). Exploratory pairwise comparisons suggested higher heart weight relative to body weight in XY T250 mice compared with XY placebo and Tfm T250; however, this did not remain significant in the overall ANOVA.

To determine whether these structural changes were accompanied by activation of hypertrophic signalling pathways, the myocardial expression of established cardiac remodelling markers was examined by quantitative PCR (Figure 3E). Supraphysiological testosterone treatment significantly increased expression of Nppa, Nppb and Acta1 in XY littermates compared with XY placebo controls (all *p* < 0.05), while expression of the anti-hypertrophic regulator Gsk3b was significantly reduced (*p* < 0.05). In contrast, expression of Myh7 and Tagln was unaltered by testosterone treatment. No significant differences in the expression of any hypertrophy-associated genes were observed between treatment groups in AR-deficient Tfm mice.

## 4. Discussion

The present study demonstrates that testosterone exerts distinct cardiovascular effects that are highly dependent on tissue context, AR signalling and hormone exposure level. While supraphysiological testosterone attenuated early atherogenic changes within the vasculature, excessive androgen exposure promoted early myocardial remodelling through an AR-dependent mechanism. These findings highlight that cardiovascular responses to testosterone are not uniform, but instead reflect divergent cellular programmes regulating vascular homeostasis and myocardial adaptation.

Consistent with previous observations, testosterone markedly reduced diet-induced aortic fatty streak formation both with and without the presence of functional AR signalling over 28 weeks [22,28,30,31]. Consistent with the established pro-atherogenic phenotype of AR-deficient Tfm mice fed an atherogenic diet, placebo-treated Tfm animals exhibited significantly greater fatty streak formation than AR-intact XY littermates, confirming the importance of endogenous androgen signalling in maintaining vascular homeostasis [22,28]. The effects of testosterone on atherosclerotic disease remain an area of ongoing debate. While our findings demonstrate reduced aortic atherosclerotic burden following testosterone exposure in this model, clinical imaging data from the Testosterone Trials reported increased coronary plaque volume following testosterone therapy in older men with hypogonadism [32]. However, the clinical implications of changes in plaque volume remain uncertain, as alterations in plaque size do not necessarily reflect changes in plaque composition, stability, or vascular function. Indeed, remodelling of the atherosclerotic plaque, including changes in immune cell infiltration and extracellular matrix composition, may influence disease progression or regression independently of overall plaque burden. Further studies integrating structural, functional, and molecular assessments of the vascular wall are required to fully understand the effects of testosterone on atherosclerotic disease.

In this study, atheroprotection is maintained following sustained supraphysiological testosterone exposure. Importantly, increasing testosterone exposure beyond physiological replacement failed to produce further reductions in aortic lipid deposition with and without functional AR, suggesting that the vascular actions of testosterone are not simply dose dependent. The absence of additional vascular benefit with supraphysiological testosterone in AR-intact mice suggests that restoration of physiological androgen signalling is sufficient to maintain vascular homeostasis, particularly when lesion formation is already minimal.

The persistence of atheroprotection following concurrent administration of either anastrozole or fulvestrant further suggests that the vascular actions of supraphysiological testosterone do not require classical nuclear AR or oestrogen receptor signalling via aromatisation to 17β-oestradiol. This contrasts with previous work where the AR-independent beneficial effects of physiological testosterone were shown to be at least partly through oestrogen receptor actions as anastrozole or fulvestrant increased lipid deposition compared with testosterone alone in Tfm mice [28]. Nathan et al. [33] demonstrated that testosterone reduced atherosclerotic lesion formation in LDL receptor-deficient mice, an effect that was abolished by concomitant administration of the aromatase inhibitor anastrozole, supporting a key role for local aromatisation of testosterone to 17β-oestradiol in mediating its anti-atherogenic effects. 

Although the precise molecular pathways were not investigated, the results are consistent with an increasing body of evidence demonstrating rapid non-genomic actions of testosterone within the vascular wall [34]. Testosterone has been shown to regulate endothelial nitric oxide bioavailability, intracellular calcium signalling, vascular smooth muscle ion channel activity and inflammatory responses through membrane-associated signalling pathways that operate independently of nuclear AR activation [35,36,37,38]. In parallel, testosterone has been reported to influence macrophage phenotype, lipid uptake and cholesterol efflux, cellular processes that are central to the metabolic regulation of early atherogenesis [1,39,40,41]. While these mechanisms remain speculative in the context of the present study, they provide biologically plausible pathways through which testosterone may preserve vascular metabolic homeostasis and limit fatty streak formation without requiring classical nuclear AR or oestrogen receptor signalling.

Additional studies and reviews have further highlighted the complexity of androgen signalling within the vasculature, suggesting that both aromatase-derived oestrogen signalling and direct AR activation contribute to vascular homeostasis in a context- and cell-specific manner [4,42,43].

In contrast to the vascular effects, supraphysiological testosterone promoted structural and molecular features consistent with early cardiac hypertrophic remodelling exclusively in AR-intact XY littermates, indicating that myocardial remodelling requires intact AR signalling. Absolute heart weight, cardiomyocyte cross-sectional area and myocardial expression of the hypertrophic markers Nppa, Nppb and Acta1 were all significantly increased following supraphysiological testosterone treatment, whereas expression of the anti-hypertrophic regulator Gsk3β was significantly reduced. None of these changes were evident in AR-deficient Tfm mice, demonstrating that the myocardial response to supraphysiological testosterone requires intact AR signalling. Although the increase in heart weight was attenuated following normalisation to body weight, the accompanying increase in cardiomyocyte size together with activation of a hypertrophic transcriptional programme supports the interpretation that supraphysiological testosterone initiates early AR-dependent cardiac remodelling rather than established pathological hypertrophy. Increased expression of Nppa and Nppb, encoding atrial and brain natriuretic peptides, respectively, represents a recognised molecular hallmark of the foetal gene programme that is reactivated during pathological cardiac hypertrophy and is regulated by pathways including calcineurin/NFAT and GATA4 signalling [44]. Increased Acta1 expression reflects cytoskeletal remodelling associated with cardiomyocyte growth and remodelling of the cardiomyocyte contractile apparatus [45]. Conversely, reduced myocardial Gsk3β expression is consistent with diminished anti-hypertrophic signalling, as GSK3β functions as a constitutive negative regulator of pathological cardiac growth [46]. The absence of changes in Myh7 indicates that supraphysiological testosterone did not induce the full foetal contractile switch associated with pathological hypertrophy, while unchanged Tagln expression suggests limited activation of fibrosis-associated remodelling. 

Interestingly, several of the molecular changes identified extend beyond structural remodelling and have recognised roles in the regulation of cellular metabolism. GSK3β represents a central metabolic kinase linking insulin signalling, glycogen metabolism and protein synthesis with cardiac growth [47,48], while natriuretic peptides encoded by *Nppa* and *Nppb* are increasingly recognised as endocrine regulators of mitochondrial function, lipid oxidation and systemic energy homeostasis [49,50]. Although these metabolic pathways were not directly investigated in the present study, the observed transcriptional changes are consistent with the concept that AR-dependent cardiac remodelling is accompanied by coordinated metabolic adaptation to support increased energetic demand.

Together, these molecular alterations may indicate an early stage of AR-dependent cardiac growth, potentially accompanied by metabolic adaptation to increased energetic demand, as androgen signalling has been shown to regulate cardiomyocyte glucose metabolism and hypertrophic growth pathways [51]. Although previous experimental studies have demonstrated that supraphysiological testosterone can promote cardiomyocyte hypertrophy, many have relied on pharmacological androgen administration in wild-type models, making it difficult to distinguish direct AR-mediated effects from indirect endocrine or metabolic influences. Importantly, the absence of structural or molecular evidence of hypertrophic remodelling in Tfm mice in the present study provides mechanistic evidence that AR signalling is required to couple supraphysiological testosterone exposure to the structural and transcriptional adaptations characteristic of early cardiac remodelling. 

These findings may also provide mechanistic insight into observations from individuals using supraphysiological anabolic-androgenic steroids, in whom prolonged exposure has been associated with pathological cardiac remodelling, impaired left ventricular function and increased risk of cardiomyopathy. Although our experimental model does not directly replicate anabolic steroid abuse, the data support the concept that excessive androgen exposure may induce cardiac adaptations distinct from those observed with physiological testosterone replacement [52,53].

The contrasting vascular and myocardial responses observed in the present study further emphasise the tissue-specific nature of testosterone signalling within the cardiovascular system [1]. Whereas supraphysiological testosterone exerted favourable effects on early atheroma formation, myocardial remodelling occurred exclusively in AR-intact mice, indicating that individual cardiovascular tissues respond to androgen exposure through distinct molecular mechanisms. Such tissue specificity is increasingly recognised as a fundamental characteristic of steroid hormone biology and is likely to reflect differences in AR signalling, intracellular signalling networks, metabolic requirements and cellular phenotype between endothelial cells, vascular smooth muscle cells and cardiomyocytes [4]. Collectively, these findings suggest that optimisation of testosterone therapy should seek to restore physiological androgen signalling sufficient to maintain vascular and metabolic homeostasis while avoiding unnecessary androgen excess that may differentially engage AR-dependent myocardial remodelling without conferring additional vascular benefit. Clinically, this reinforces the need for careful patient selection, appropriate dose titration and regular biochemical monitoring to avoid sustained supraphysiological testosterone exposure [54,55].

The present study has several limitations that should be considered when interpreting these findings. First, the Tfm mouse provides a well-established model for investigating testosterone actions in the absence of functional AR signalling but does not fully recapitulate the complexity of testosterone deficiency or testosterone replacement therapy in humans. Second, the supraphysiological testosterone concentrations achieved in this experimental model exceed those typically targeted during clinical testosterone replacement and therefore are more appropriately considered a mechanistic model for investigating excessive androgen exposure rather than therapeutic dosing. Analytical datasets and tissue from physiologically testosterone-treated XY mice required for comparison with the present cardiac and molecular analyses were unavailable. Thus, the present study cannot determine whether physiological testosterone replacement produces comparable effects in androgen-replete XY mice. Furthermore, the assessment of early fatty streak formation provides insight into the initiation of atherogenesis but does not address later stages of plaque progression or stability. Similarly, although the present study identified structural and molecular features consistent with early cardiac hypertrophic remodelling, functional cardiac assessment was not performed, and therefore the long-term physiological significance of these changes remains to be established. While transcriptional alterations suggested metabolic adaptations accompanying myocardial remodelling, direct assessment of mitochondrial function, substrate utilisation or metabolic flux was not undertaken. Finally, while the findings support distinct cellular mechanisms underlying the vascular and myocardial actions of testosterone, the downstream signalling pathways responsible for these divergent tissue responses were not directly investigated. Future studies should combine transcriptomic, proteomic and metabolic approaches with longitudinal assessment of vascular, myocardial and haematological outcomes to further define the tissue-specific mechanisms through which testosterone influences cardiometabolic health.

## 5. Conclusions

In conclusion, this study demonstrates that sustained supraphysiological testosterone exposure produces divergent cardiovascular effects through tissue-specific mechanisms. While testosterone attenuated early aortic fatty streak formation, excessive androgen exposure promoted early cardiac hypertrophic remodelling exclusively in AR-intact mice, demonstrating that myocardial growth responses require functional AR signalling in vivo. These findings highlight that testosterone does not exert a uniform cardiovascular effect, but instead activates distinct cellular programmes governing vascular homeostasis and myocardial adaptation.

Importantly, the absence of hypertrophic remodelling in AR-deficient Tfm mice provides direct genetic evidence that supraphysiological testosterone-induced cardiac growth is AR-dependent, extending previous observations from wild-type models. Collectively, these findings support the concept that physiological androgen signalling may be sufficient to maintain vascular and metabolic homeostasis, whereas excessive androgen exposure preferentially engages myocardial growth pathways associated with increased cellular and energetic demand. These observations reinforce the importance of maintaining physiological testosterone concentrations during therapy to maximise therapeutic benefit while minimising tissue-specific consequences of androgen excess.

## Data Availability

The raw data supporting the conclusions of this article will be made available by the authors on request.

## Abbreviations

The following abbreviations are used in this manuscript:

ANOVA	Analysis of variance
AR	Androgen receptor
Cavg	Time-average concentration
CSA	Cross-sectional area
ERα	Oestrogen receptor-α
H&E	Haematoxylin and eosin
ORO	Oil Red O
SEM	Standard error of the mean
Tfm	Testicular feminised
TRAVERSE	The Testosterone Replacement Therapy for Assessment of Long-term Vascular Events and Efficacy Response in Hypogonadal Men
TTh	Testosterone therapy
